# The Sclerometrical, Mechanical, and Wear Behavior of Mg-Y-Nd Magnesium Alloy after Deep Cryogenic Treatment Combined with Heat Treatment

**DOI:** 10.3390/ma14051218

**Published:** 2021-03-04

**Authors:** Adrian Barylski, Krzysztof Aniołek, Grzegorz Dercz, Marian Kupka, Izabela Matuła, Sławomir Kaptacz

**Affiliations:** Faculty of Science and Technology, Institute of Materials Engineering, University of Silesia in Katowice, 41-500 Chorzów, Poland; krzysztof.aniolek@us.edu.pl (K.A.); grzegorz.dercz@us.edu.pl (G.D.); marian.kupka@us.edu.pl (M.K.); imatula@us.edu.pl (I.M.); slawomir.kaptacz@us.edu.pl (S.K.)

**Keywords:** Mg-Y-Nd magnesium alloy, deep cryogenic treatment (DCT), microstructure, X-ray diffraction, microhardness, sclerometrical properties, wear

## Abstract

The paper investigates changes in the structure, microhardness, and sclerometrical and tribological properties of a Mg-Y-Nd alloy under the influence of deep cryogenic treatment (DCT) in combination with heat treatment. The solution treatment was carried out at 545 °C for 8 h, aging was carried out at 250 °C for 24 h, and the deep cryogenic treatment applied at different treatment stages was performed at −196 °C. Tests showed a significant increase in the number of β-phase precipitates identified as Mg_46.1_Y_6.25_RE_3.45_ in the alloy subjected to DCT after solution treatment followed by aging. In addition, an approximately 20% reduction of the grain size was observed. Changes in the structure in the precipitation process strengthened the alloy and resulted in an increase of its hardness. At the same time, sclerometric tests allowed the micromechanism of wear and the coefficient of resistance to abrasive wear to be determined. Tribological tests showed a three-fold reduction in the volumetric wear and a considerable reduction of the friction coefficient, with the main mechanism observed during friction being abrasive wear. The most favorable properties of the alloy were obtained after precipitation hardening combined with DCT, resulting in a large increase in resistance to abrasive wear. Additionally, the formation of deep scratches in the examined material was reduced. The introduction of sub-zero treatment reduces the precipitation hardening time, and the results obtained indicate that the service life of the Mg-Y-Nd alloy can be extended.

## 1. Introduction

As they have the lowest density (about 1.8 g/cm^3^) among engineering materials, as well as high specific strength, castability, easy machinability, good weldability, and recyclability, magnesium alloys are used in a number of industries (military, automotive, aviation) [1,2,3,4]. They are also frequently applied as biomaterials due to their favorable Young’s modulus values and their compressive and tensile strengths in relation to the properties of cortical bone. This are their advantages over the materials used in orthopedics to date, such as austenitic steels, NiTi alloys, or titanium alloys. The high rigidity of these materials slows down fracture healing processes and bone tissue stimulation due to the reduction of natural bone load, which significantly prolongs the patient’s convalescence [5,6,7,8,9]. For biomedical applications, magnesium–rare earth alloys (WE43, WE54) are proposed among other materials [10,11,12,13,14]. Magnesium is also commonly applied as a component of other alloys and can be used for doping of nanocrystalline materials (e.g., nanocrystalline aluminum), improving their strength by preventing nucleation and propagation of intercrystalline cracking [15].

Despite having many advantages, magnesium alloys also have numerous limitations due to their low plasticity, high chemical reactivity, poor corrosion resistance, high tribological wear, and high production costs [3].

The yttrium content in magnesium alloys leads mainly to refinement of the microstructure and to the formation of new phases, as presented in [16]. The addition of rare earth elements (RE), which form solid solutions with limited solubility, to magnesium alloys allows hardening through precipitation, which is one of the effective methods for improving their properties. In the precipitation process, Guinier-Preston zones are formed, which transform into coherent hexagonal precipitates *β*″ and partly coherent precipitates *β*′, and after a properly selected aging time, into a non-coherent *β*-phase. The formation of precipitates results in strengthening of the alloy [17,18,19,20,21,22,23,24]. The aging process is also used for other engineering materials, with improved bending strength of 3D-printed polymer composites aged below the glass transition temperature being observed [25].

There is also a growing worldwide interest in deep cryogenic treatment (DCT) conducted in liquid nitrogen (−196 °C). DCT has positive effects, such as increased resistance to tribological wear, improved mechanical properties, and reduced material stress.

However, previous studies have mainly involved steel [26,27,28,29,30] and magnesium alloys with aluminum [31,32,33,34] and gadolinium [35,36,37,38]. The main effect of deep cryogenic treatment in the case of non-ferrous metal alloys forming systems with limited solubility of the other component is an increase in the number of nucleation sites of the secondary phase particles, and thus an increase in the number of precipitates [39]. The precipitates formed significantly strengthen magnesium alloys [24], as demonstrated in our previous paper, and reduce their wear [18].

The aim of this study was to investigate the effect of combining deep cryogenic treatment with precipitation hardening and the possibility of improving the sclerometric, mechanical, and tribological properties of the Mg-Y-Nd magnesium alloy.

## 2. Materials and Methods

Materials and Equipment

The research object consisted of a magnesium alloy with rare earths (RE), Mg-Y-Nd (WE54), supplied in the form of rods by Luxfer MEL Technologies (former Magnesium Elektron, Manchester, England). Disc samples with a rod diameter of 25.4 mm and thickness of 5 mm were prepared for the tests. In the as-delivered condition, the alloy had the following composition, as confirmed by chemical analysis (Y = 5.2; Nd = 1.6; Zr = 0.5; RE = 2.6, Mg = residue wt.%).

Precipitation hardening (solution treatment and aging) was performed in an FCF-5M laboratory muffle furnace (Czylok, Jastrzębie-Zdrój, Poland) at temperatures of 545 °C (solution treatment) and 250 °C (aging). Deep cryogenic treatment was conducted after different phases of heat treatment in liquid nitrogen (−196 °C) over a period of 24 h. The heat treatment parameters were selected based on our previous research [19]. Detailed data concerning all investigated variants and heat treatment durations are presented in Table 1.

The preparation of samples (Metalog Guide A procedure) consisted of grinding using 320–2000 grit abrasive paper and polishing. Microsections for metallographic tests were etched in a 3% HNO_3_/ethyl alcohol solution. Specimens for tribological tests had a uniform surface roughness *Ra* = 0.13 µm, and before each test they were cleaned in an ultrasonic washer using acetone.

Observation of the metallographic specimens and a quantitative description of their structures (average grain area *A* (μm^2^), size *G* (ASTM E112) [40], volume fraction of intermetallic phases *V_V_* (%)) were performed using an OLYMPUS GX-51 (Olympus, Tokyo, Japan) light microscope equipped with a camera and Stream Essentials software. The specimens were also observed by means of a JEOL JSM-6480 (Jeol, Tokyo, Japan) scanning electron microscope equipped with an adapter for X-ray microanalysis using the Energy-dispersive X-ray spectroscopy-EDS method (IXRF, Austin, TX, USA).

The phase content of the WE54 magnesium alloy was examined using X-ray diffraction (XRD) using a Philips X’Pert diffractometer (Philips, Almelo, Holland) fitted with a copper anode tube (Cu *Kα λ* = 1.54178 Å). Scanning was performed with a 0.04 step and a counting time of 25 s/step, in the angular range of 10°–140° 2θ.

Microhardness of the Mg-Y-Nd alloy was examined at a load of 500 gf (4.9 N) using a diamond Vickers indenter with a 138° cone angle on a Wolpert Wilson Instruments tester model 401 MVD (Wolpert Wilson, Worcester, MA, USA). The hold time under maximum load was 15 s. The result of each measurement was the average of 10 indents.

Sclerometrical tests were performed with a Micron-Gamma device (manufactured by The National Technical University of Ukraine, Kiev), using a Rockwell indenter with a 200 µm radius. On each specimen, 3 scratches were made under different loads: F_n_ = 1 N, 2 N and 4 N. The scratches had a length of ca. 4.5 mm; the scratching rate was 5.4 mm/min. The micromechanism of abrasive wear *β* and the abrasive wear resistance coefficient *K_αβ_* were calculated based on profilographometric measurements of the furrow area *A* and the plastic elevation *B* of the scratches formed during the scratch test using a Form Talysurf Series 2-50i profilometer (Taylor-Hobson, Leicester, England), in accordance with Equations (1) and (2) [41]:

(1) $$\beta =\frac{1}{n}\overset{n}{\underset{i=1}{\sum }}\frac{A_{i}-B_{i}}{A_{i}}$$

where *A_i_* = furrow area, *B_i_* = elevation area (where *β* = 0, ideal microploughing takes place without material removal; where *β* = 1, ideal microcutting takes place without forming ridges), and [42,43]:

(2) $$K_{\alpha \beta }=\beta \frac{A_{i}\cdot HV}{F_{n}}$$

where *β* = abrasive wear micromechanism, *A_i_* = furrow area, *HV* = Vickers hardness, *F_n_* = normal force during the scratch test.

The tracks that formed during the scratch test were illustrated by acquiring an isometric 3D image together with a color 2D change map using the TalyMap Universal and Matlab software programs [44,45]. A scratch area of 1.5 mm × 5 mm was examined, maintaining the following sampling distance: x = 1 μm, y = 30 μm, z = 16 nm.

**Figure 1:**
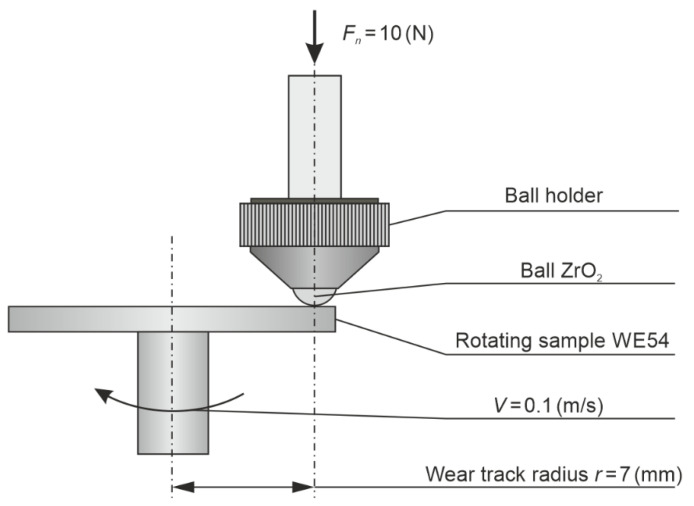
Conditions of tribological tests used for the investigated WE54 alloy.

Tribological tests (ball-on-disc) in rotational motion were performed using a TRN device with the InstrumX software (Anton Paar, Corcelles-Cormondrèche, Switzerland) with the conditions specified in Figure 1.

Each time, 4 repetitions were performed over a 100 m friction distance. The counter-specimens were ZrO_2_ balls measuring 6 mm in diameter. The wear was examined under dry friction conditions, as recommended by the VAMAS (Versailles Project on Advanced Materials and Standards) technical note and in compliance with the ASTM G99 standard [46,47]. The following parameters were determined: mass wear *M_w_*, volumetric wear *V_w_*, linear wear *L_W_*, and mean friction coefficient *µ_mean_*. The wear traces, similarly to the scratches, were visualized in 3D using a Taylor-Hobson profilometer (Leicester, England). A wear track area of 2.5 mm × 5 mm was examined, maintaining the following sampling distance: x = 1 μm, y = 10 μm, z = 16 nm.

Profilographometric measurements also determined the average area of the wear trace *P*, which was necessary to calculate the volumetric wear (3) [48]:

(3) $$V_{W}=\frac{V}{F_{n}\cdot s}\left({\mathrm{mm}}^{3}/\mathrm{Nm}\right)$$

where *F_n_ =* the load applied, *s =* friction distance, *V =* volume of the disc wear track (=P·2πr)

## 3. Results and Discussion

### 3.1. Changes in the Microstructure of the Mg-Y-Nd Alloy as a Result of Deep Cryogenic Treatment

**Figure 2:**
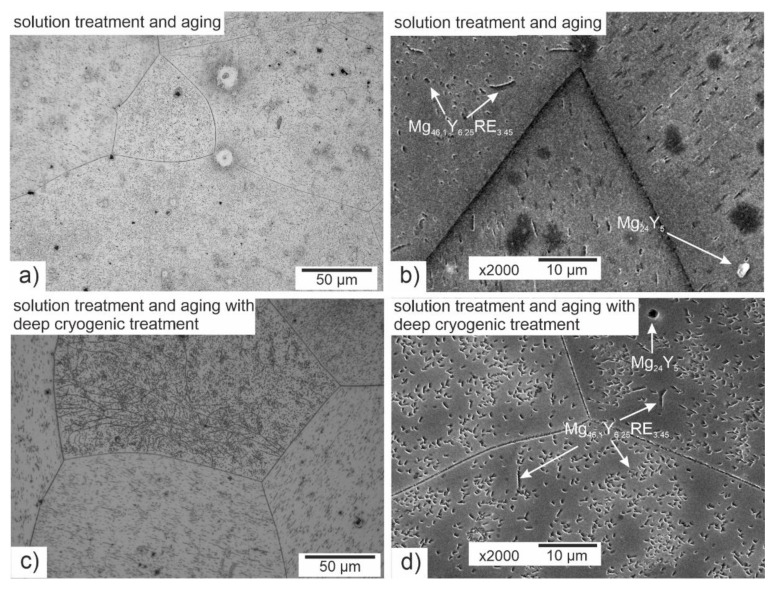
Microstructure of the Mg-Y-Nd alloy after precipitation hardening (solution treatment at 545 °C/8 h and aging at 250 °C/24 h) (**a**,**b**) and after precipitation hardening with a deep cryogenic treatment (**c**,**d**).

The microstructure of the investigated magnesium alloy after precipitation hardening and after combining the latter with DCT is presented in Figure 2.

**Figure 3:**
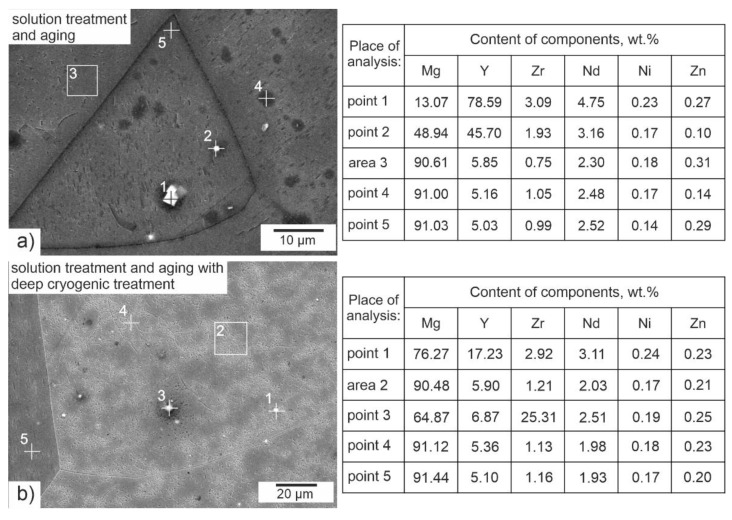
X-ray microanalysis using the EDS method for the Mg-Y-Nd alloy after precipitation hardening (**a**) and after precipitation hardening with a deep cryogenic treatment (**b**).

**Figure 4:**
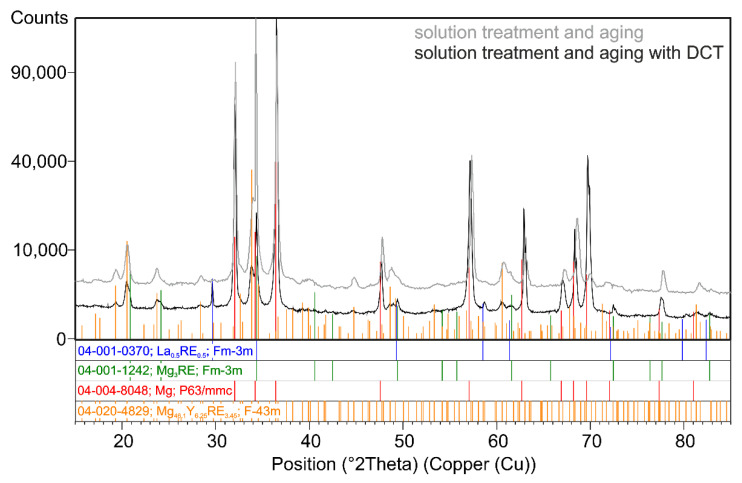
X-ray diffraction pattern (XRD) of the Mg-Y-Nd alloy after precipitation hardening (**a**) and after precipitation hardening with a deep cryogenic treatment (**b**).

**Figure 5:**
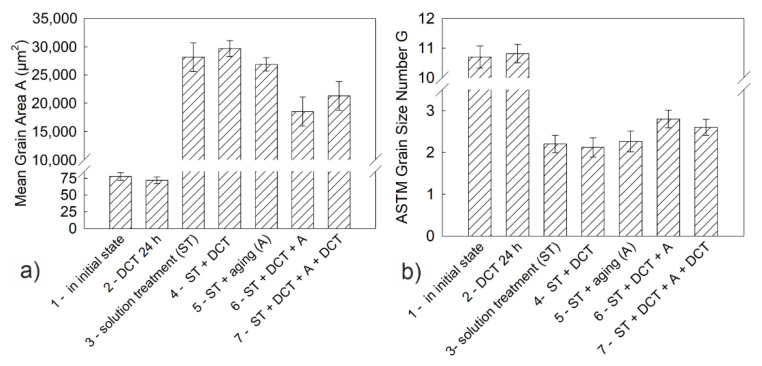
The average grain area (**a**) and G grain size number (**b**) of the Mg-Y-Nd alloy (as per ASTM E112 standard) in the initial state and after different treatment variants in combination with DCT.

**Figure 6:**
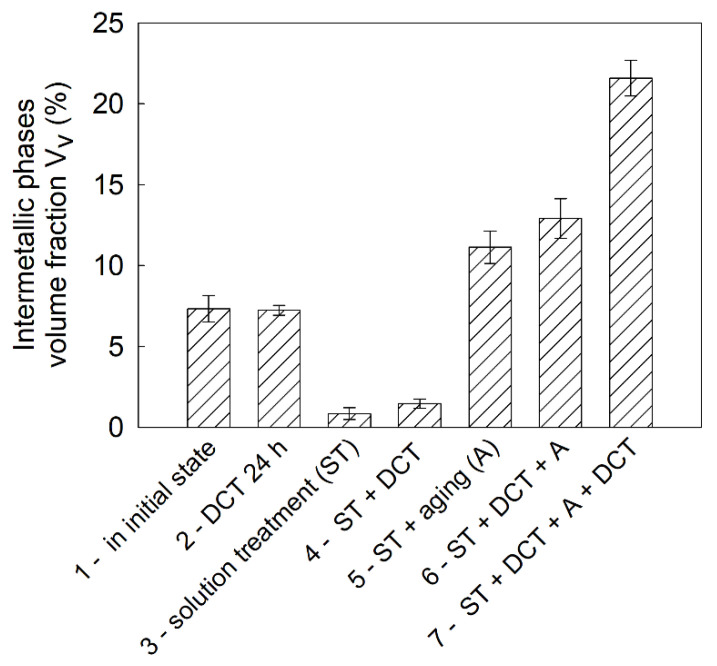
The average volume fraction of the intermetallic phases of the Mg-Y-Nd alloy (as per ASTM E112 standard) in the initial state and after different treatment variants in combination with DCT.

The results of the chemical composition microanalysis (EDS) of the magnesium alloy after precipitation hardening without and with DCT are presented in Figure 3. Examples of diffraction patterns obtained for the Mg-Y-Nd alloy after the applied treatment are presented in Figure 4. The grain size and volume fraction of intermetallic phases are presented in Figure 5 and Figure 6.

Observation of the microstructure by means of an optical and electron microscope, backed up with examination of the phase composition and analysis of the grain size and volume fraction of intermetallic phases, corroborated the great influence of deep cryogenic treatment on the precipitation process of the Mg-Y-Nd alloy observed during heat treatment. After conventional heat treatment (precipitation hardening), precipitates of phase *β* occurred in the alloy microstructure, which were identified as Mg_46.1_Y_6.25_RE_3.45_. Their average volume fraction was 11.13% and the grain size was approximately 27,000 µm^2^. The additional introduction of deep cryogenic treatment (DCT) to heat treatment considerably increased the share of phase *β*; the average volume fraction *V_v_* grew twice up to 21.58%. Moreover, a 20% reduction of the grain size to 18,000–21,000 µm^2^ was observed. The increase in the number of new precipitates in the examined magnesium alloy after sub-zero treatment can be explained by the formation of new nucleation sites as a result of volumetric contraction, generation of high stresses, and storage of deformation energy, as well as a reduction in the parameters of the alloy’s crystal lattice. Similar observations were described in Sonar’s work regarding cryogenic treatment of metals and alloys [27]. Deep cryogenic treatment makes it possible to reduce the time needed to obtain a similar number of precipitates by half as compared to an alloy subjected to the heat treatment itself, and also reduces the grain growth, which has an adverse effect on the wear and properties of a magnesium alloy containing rare earth metals [18,49].

### 3.2. Micromechanical and Sclerometrical Examination of the Mg-Y-Nd Alloy

**Figure 7:**
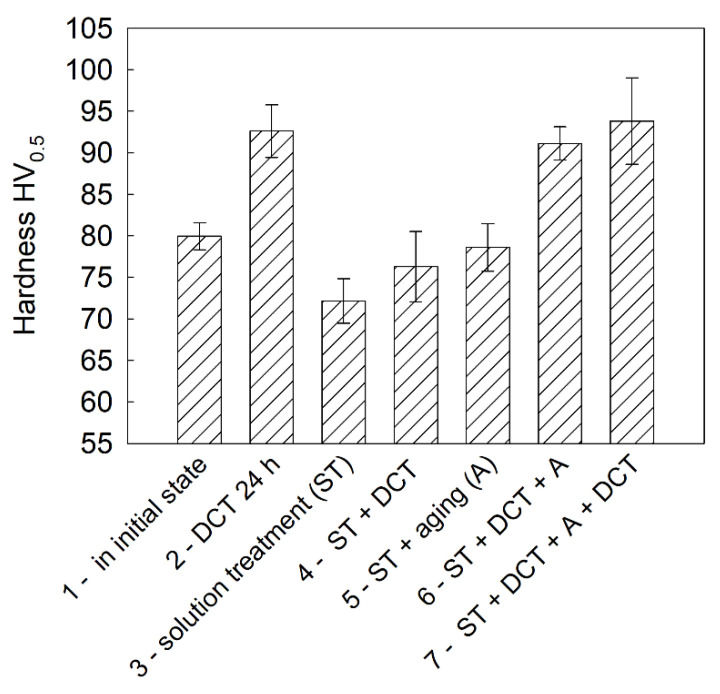
Changes in Vickers microhardness values of the Mg-Y-Nd alloy in the initial state and after different heat treatment variants in combination with DCT.

The deep cryogenic treatment and the changes in the structure it induces affect both the mechanical and sclerometrical properties of a magnesium alloy containing rare earth metals. Figure 7 shows the changes in Vickers microhardness values.

In its initial state, the WE54 alloy had a hardness of approximately 80 HV, which was in compliance with the manufacturer’s certificate. After the solution treatment, a reduction in hardness was observed, which was connected mainly with the dissolution of primary precipitates and a large increase in the grain size. The application of deep cryogenic treatment, especially when combined with heat treatment, effectively increased the microhardness of the alloy to about 94 HV. It is also worth mentioning that cryogenic treatment itself also resulted in an approximately 16–20% increase in alloy hardness. Changes in the hardness of the alloy were mainly influenced by changes in the precipitation process. A larger number of *β*-phase precipitates enhanced the effect of precipitation hardening. Similar effects were observed for magnesium alloys with aluminum [34] or gadolinium [35] additions. The decrease in grain size observed in the present study also induced an increase in the microhardness of the investigated alloy containing rare earth metals.

**Figure 8:**
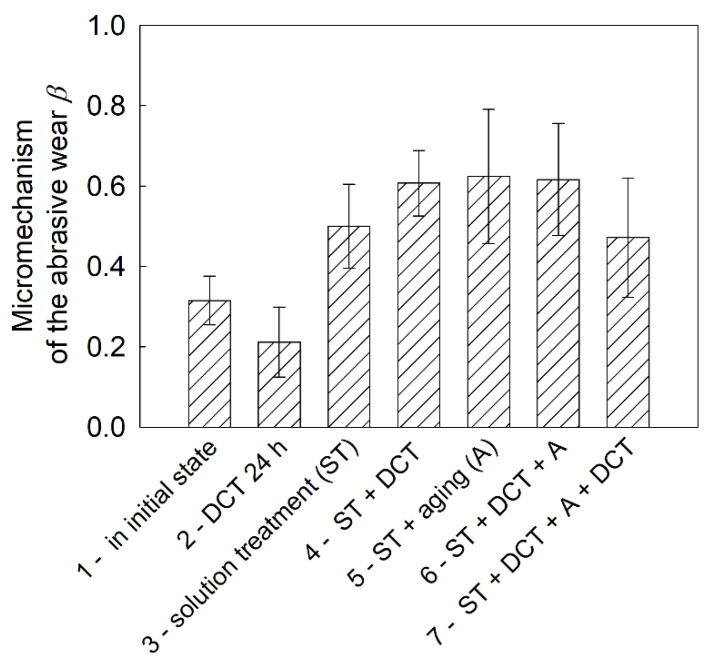
Changes in the wear micromechanism of the Mg-Y-Nd alloy in the initial state and after different heat treatment variants in combination with DCT.

**Figure 9:**
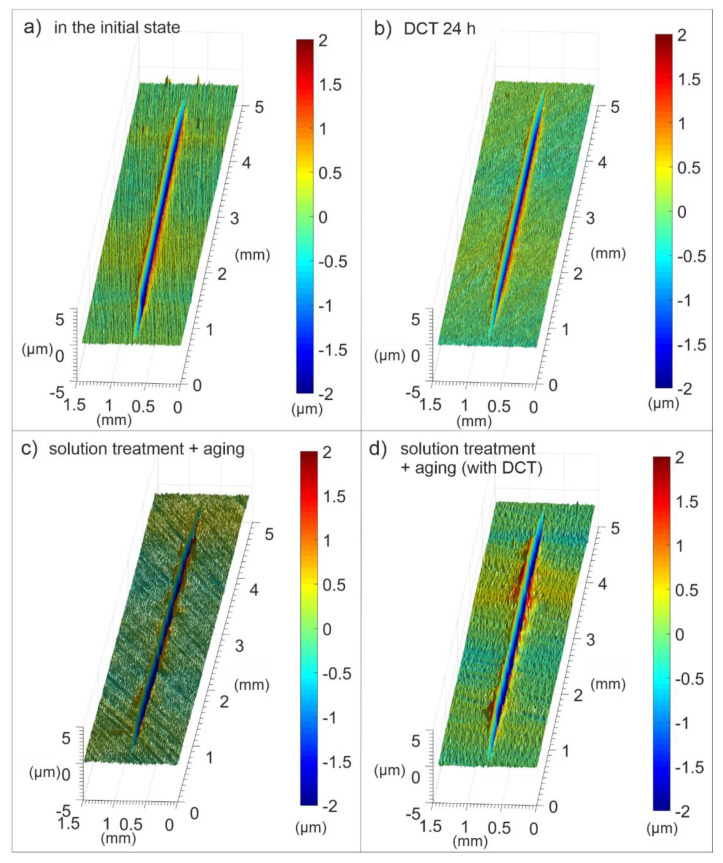
Isometric images (3D) of scratch wear tracks of the Mg-Y-Nd alloy in the initial state and after different heat treatment variants: in initial state (**a**) and after deep cryogenic treatment (**b**), solution treatment + aging (**c**), solution treatment + aging + DCT (**d**).

**Figure 10:**
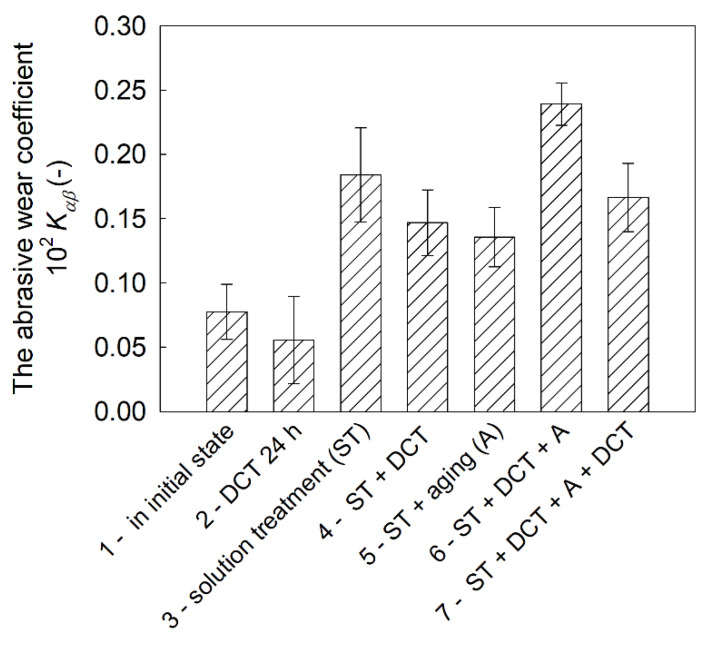
Changes in the abrasive wear resistance coefficient of a magnesium alloy containing rare earth metals in the initial state and after different heat treatment variants combined with DCT.

Sclerometric tests of the Mg-Y-Nd alloy after different heat treatments in combination with cryogenic treatment (Figure 8 and Figure 9) made is possible to determine the *β* coefficient, which determines the micromechanism of abrasive wear, as well as the abrasive wear resistance coefficient, *K_αβ_* (Figure 10) [41,42,43]. Both of these parameters, according to the methodology described in the previous part of the paper, depend on the furrow area, A, and the elevation area, *B*, of scratch traces, while in the case of the *K_αβ_* coefficient, they also depend on the hardness of the material.

From the analysis of the results presented in Figure 8, it can be concluded that the discussed magnesium alloy has a mixed wear mechanism (so-called extrusion, or in other words, scratching 0 < *β* < 1), which meant that the material was partially deformed and partially cut during the scratch test. It can be noted, however, that the wear micromechanism of the alloy in the initial state and after the solution treatment was characterized by a predominance of microploughing, *β*→0. On the other hand, the modification of mechanical properties, and in particular the increase in hardness caused by heat treatment in combination with cryogenic treatment, caused changes in the micromechanism of wear towards microcutting, *β*→1. This meant that a smaller part of the alloy was elevated when trying to scratch the surface, which can also be observed in 3D stereometric images (Figure 9).

The abrasive wear resistance coefficient, *K_αβ_* (Figure 10), which is calculated based on stereometric measurements of scratch traces, allows the conclusion that a material subjected to heat treatment (solution treatment, aging) in combination with deep cryogenic treatment (DCT) has the most favorable resistance to abrasive wear. This should translate directly into the behavior of the alloy during tribological tests.

### 3.3. Tribological Examination of the Mg-Y-Nd Alloy

**Figure 11:**
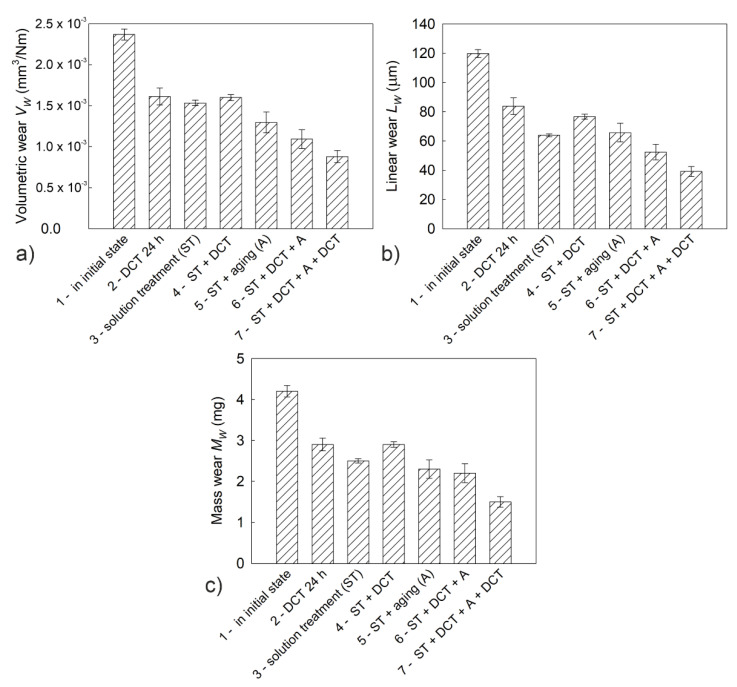
Volumetric wear (**a**), linear wear (**b**), and mass wear (**c**) of the Mg-Y-Nd alloy in the initial state and after different heat treatment variants in combination with DCT.

**Figure 12:**
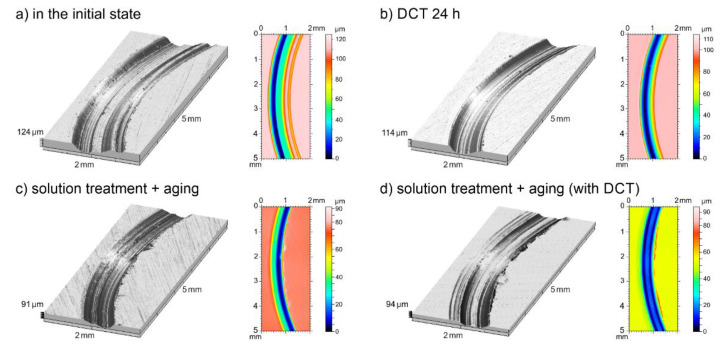
Isometric (3D) and color map (2D) images of wear tracks of the Mg-Y-Nd alloy in initial state (**a**) and after deep cryogenic treatment (**b**), solution treatment + aging (**c**), solution treatment + aging + DCT (**d**).

**Figure 13:**
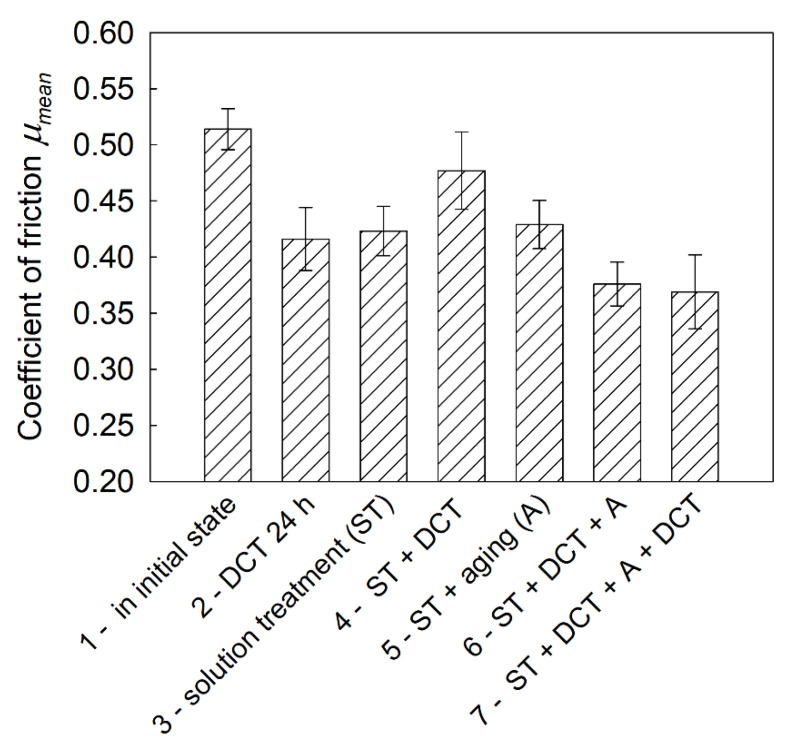
Stabilized friction coefficient of the Mg-Y-Nd alloy in the initial state and after different heat treatment variants in combination with DCT.

**Figure 14:**
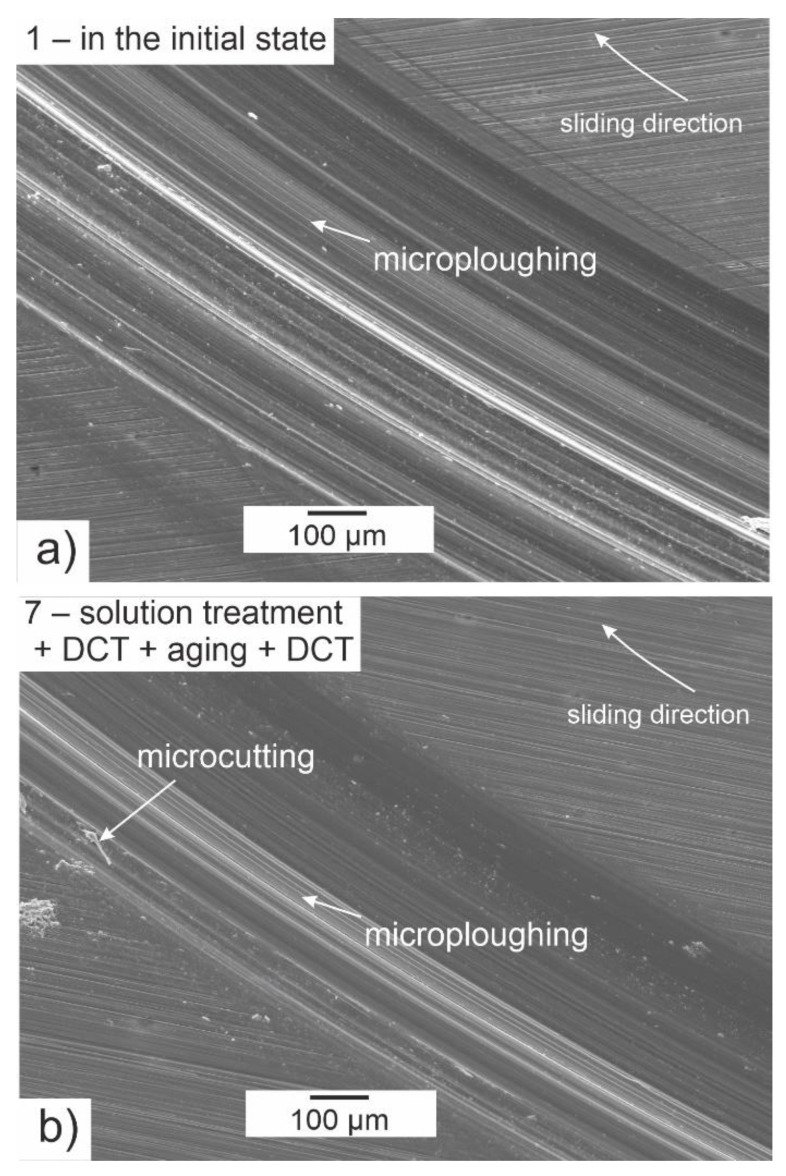
SEM images after tribological tests in rotational motion of the Mg-Y-Nd alloy in the as-delivered condition (**a**) and after heat treatment combined with DCT (**b**).

Deep cryogenic treatment combined with a solution treatment and aging, regardless of changes in hardness and sclerometrical properties, also had a significant influence on the tribological properties of the investigated alloy. The volumetric wear *V_w_*, mass wear *M_w_*, and linear wear *L_w_* are presented in Figure 11a–c; changes in the stabilized friction coefficient *μ_mean_*; 3D isometric images and SEM images of the wear surfaces are presented in Figure 12, Figure 13 and Figure 14.

Analysis of the results of tribological tests in rotational motion (Figure 11) showed that the discussed alloy in the initial state had a low resistance to abrasive wear. Its volumetric wear *V_w_* = 2.37 × 10^−3^ mm^3^/Nm, its linear wear *L_w_* = 119.73 μm, and its mass wear *M_w_* = 4.2 mg. The application of deep cryogenic treatment only caused reductions in all examined parameters by about 25–30%. However, the combination of deep cryogenic treatment with heat treatment (solution treatment and aging) causes reductions of volumetric wear by 2.5-fold, of linear wear by 3-fold, and of mass wear by almost 3-fold. These results indicate a significant improvement in the service life of Mg-Y-Nd alloy subjected to the described heat treatment.

The improvement of tribological properties was connected mainly with the formation of a much larger number of *β*-phase precipitates (Mg_46.1_Y_6.25_RE_3.45_) in the alloy subjected to sub-zero treatment in combination with additional heat treatment. The precipitates in the form of lamellae modified the micromechanical and sclerometrical properties of the alloy, leading to a three-fold improvement in its tribological properties in comparison with the alloy in its initial state. This is consistent with Archard’s law, which says that resistance to abrasive wear depends on the conditions and properties of the material [49], and is also consistent with the research results discussed in previous studies [50,51]. The above results are also confirmed in graphical form as selected 3D isometric images and color map 2D images of the surfaces after tribological tests (Figure 12). The wear traces after the proposed treatment were mainly characterized by a reduction in their width and depth.

During the tribological tests, changes in the friction coefficient were also recorded. Figure 13 presents the effects of the proposed heat treatment on the stabilized friction coefficient, *μ_mean_*. As the tribological tests showed, one of the reasons for the substantial wear of the Mg-Y-Nd alloy in its initial state was the high value of the friction coefficient, *μ_mean_* = 0.51.

The most advantageous results, similarly to the earlier analyzed parameters, were obtained for the alloy subjected to deep cryogenic treatment combined with a solution treatment and aging. The stabilized friction coefficient was reduced by about 30% to *μ_mean_* = 0.37, as compared to the initial state.

Images of the wear tracks obtained from a scanning electron microscope (Figure 14) also show a substantial reduction in wear of the alloy after the proposed treatment. It can be noticed in the pictures that the main mechanism of wear was abrasive wear, characterized by the formation of numerous grooves and cavities (microploughing) parallel to the direction of sliding. As in previous studies [37,38,39], small areas of microcutting were also visible. Deep cryogenic treatment effectively reduces the formation of deep scratches, which translates directly to a reduction in tribological wear. At the same time, tribological tests did not reveal wear of the counter-specimen in the form of a ZrO_2_ ball in any of the variants tested. This must have been connected with the significant difference in hardness between the very hard balls and the Mg-Y-Nd alloy.

## 4. Conclusions

This paper investigates the structure, phase composition, hardness, and sclerometrical and tribological properties of a magnesium–rare earth alloy (Mg-Y-Nd) subjected to a solution treatment and aging in combination with deep cryogenic treatment. Based on the obtained research results and available literature data, the following conclusions can be drawn:

1. As a result of deep cryogenic treatment combined with a solution treatment and aging, a two-fold increase in the volume fraction of intermetallic phases, in particular of phase Mg_46.1_Y_6.25_RE_3.25_, took place, along with a 20% reduction in the grain size compared to precipitation hardening on its own;
2. As a consequence of changes in the microstructure, the microhardness of the investigated magnesium alloy increased, the wear micromechanism changed, and the abrasive wear resistance rate also increased;
3. Tribological tests allowed the determination of volumetric, linear, and mass wear for the alloy after different heat treatment variants in combination with DCT. It was shown that the combination of deep cryogenic treatment with precipitation hardening caused a reduction of volumetric wear *V_w_* by 2.5 times, of linear wear *L_w_* by 3 times, and of mass wear *M_w_* by almost 3 times. These results prove a significant improvement in the service life of the Mg-Y-Nd alloy;
4. Three-dimensional stereometric analysis and SEM images showed a considerable reduction of the width and depth of wear traces. The main wear mechanism was abrasion, visible in the form of numerous grooves and cavities, which formed parallel to the direction of sliding. Small areas of microploughing and microcutting were also visible;
5. The combination of DCT with precipitation hardening resulted in a 30% reduction of the stabilized friction coefficient, which also had a great influence on improving the tribological properties of the investigated alloy;
6. The main effect of the proposed treatment was the increase in the number of fine precipitates, resulting in increased precipitation hardening of the investigated alloy, and thus improved mechanical, sclerometric, and tribological properties. In this case, the precipitation process was supported by the formation of additional precipitate nucleation sites during DCT, contraction in volume, and reduction of the magnesium lattice parameter;
7. The research is being continued by the authors in order to better understand the mechanisms responsible for changes in the structure and properties of magnesium alloys containing rare earth metals subjected to complex heat treatments.

## Figures and Tables

**Table 1:** Heat treatment and deep cryogenic treatment (DCT) parameters of Mg-Y-Nd magnesium alloy.

No.	Sample Name	Solution Treatment 545 °C	DCT −196 °C	Aging 250 °C	DCT −196 °C
1	In initial state	-	-	-	-
2	DCT 24 h	-	24 h	-	-
3	Solution treatment	8 h	-	-	-
4	Solution treatment + DCT	8 h	24 h	-	-
5	Solution treatment + Aging	8 h	-	24 h	-
6	Solution treatment + DCT + Aging	8 h	24 h	24 h	-
7	Solution treatment + DCT + Aging + DCT	8 h	24 h	24 h	24 h

## Data Availability

The data presented in this study are available on request from the corresponding author.
